# USING SUPERVISED MACHINE LEARNING METHODS TO IDENTIFY INDIVIDUALS AT RISK OF DEMENTIA

**DOI:** 10.1093/geroni/igae098.1382

**Published:** 2024-12-31

**Authors:** Ayushi Divecha, Sarah Bannon, Kristen Dams-O’Connor

**Affiliations:** Icahn School of Medicine at Mount Sinai, New York, New York, United States; Icahn School of Medicine at Mount Sinai, New York, New York, United States; Icahn School of Medicine at Mount Sinai, New York, New York, United States

## Abstract

There has been an increased interest in AI and machine learning based models in early detection and diagnosis of Alzheimer’s disease (AD) and Alzheimer’s disease related dementia (ADRD). However, there is a need for feasible tools to identify individuals at risk of AD/ADRD using machine learning methods that can be validated. Early identification of risk of AD/ADRD can be valuable for building a monitoring system and planning of care. The aim of the study is to identify individuals at risk of developing dementia at baseline visit using machine learning models. Clinical and cognitive data from 5 of the largest NIH-funded studies of aging across the US (ACT, ROS, MAP, MARS, FHS) will be used to predict all-cause dementia in cognitively normal individuals at baseline using random forest models with competing risk of death. Cognitive elements like executive functioning, language, memory, visuospatial ability will be included. Since cognitive function was assessed using different measures across cohorts (MMSE and CASI), co-calibrated cognitive factor scores will be used in the trained models to allow for direct comparison of cognition across cohorts and joint analyses. We examined 3 out of 5 cohorts. From ROS/MAP/MARS cohorts, 2235 individuals qualified for the study and had a mean follow-up of 8.6 ± 5.3 years. AOC will be used to assess sensitivity and specificity of the trained models (75% training set, 25% testing set) with each of the 5 cohorts. The models will also identify features important in predicting dementia.

